# Molecular Cloning and Functional Analysis of Three *FLOWERING LOCUS T (FT)* Homologous Genes from Chinese *Cymbidium*

**DOI:** 10.3390/ijms130911385

**Published:** 2012-09-12

**Authors:** Weiting Huang, Zhongming Fang, Songjun Zeng, Jianxia Zhang, Kunlin Wu, Zhilin Chen, Jaime A. Teixeira da Silva, Jun Duan

**Affiliations:** 1 Key Laboratory of South China Agricultural Plant Genetics and Breeding, South China Botanical Garden, The Chinese Academy of Sciences, Guangzhou 510650, China; E-Mails: weitingpink@hotmail.com (W.H.); zmfang88@163.com (Z.F.); cymbidium1979@yahoo.com.cn (J.Z.); wu_kunlin@163.com (K.W.); duanj@scib.ac.cn (J.D.); 2 Graduate University of the Chinese Academy of Sciences, Beijing 100049, China; 3 Horticultural Research Institute of Guizhou Province, Guiyang 550006, China; E-Mail: chenzhilin@126.com; 4 Faculty of Agriculture and Graduate School of Agriculture, Kagawa University, Miki-cho, Kagawa 761-0795, Japan; E-Mail: jaimetex@yahoo.com

**Keywords:** *Cymbidium*, *FLOWERING LOCUS T*, cloning, gene function

## Abstract

The *FLOWERING LOCUS T* (*FT*) gene plays crucial roles in regulating the transition from the vegetative to reproductive phase. To understand the molecular mechanism of reproduction, three homologous *FT* genes were isolated and characterized from *Cymbidium sinense* “Qi Jian Bai Mo”, *Cymbidium goeringii* and *Cymbidium ensifolium* “Jin Si Ma Wei”. The three genes contained 618-bp nucleotides with a 531-bp open reading frame (ORF) of encoding 176 amino acids (AAs). Alignment of the AA sequences revealed that CsFT, CgFT and CeFT contain a conserved domain, which is characteristic of the PEBP-RKIP superfamily, and which share high identity with FT of other plants in GenBank: 94% with OnFT from *Oncidium* Gower Ramsey, 79% with Hd3a from *Oryza sativa*, and 74% with FT from *Arabidopsis thaliana*. qRT-PCR analysis showed a diurnal expression pattern of *CsFT*, *CgFT* and *CeFT* following both long day (LD, 16-h light/8-h dark) and short day (SD, 8-h light/16-h dark) treatment. While the transcripts of both *CsFT* and *CeFT* under LD were significantly higher than under SD, those of *CgFT* were higher under SD. Ectopic expression of *CgFT* in transgenic *Arabidopsis* plants resulted in early flowering compared to wild-type plants and significant up-regulation of *APETALA1* (*AP1*) expression. Our data indicates that CgFT is a putative phosphatidylethanolamine-binding protein gene in *Cymbidium* that may regulate the vegetative to reproductive transition in flowers, similar to its *Arabidopsis* ortholog.

## 1. Introduction

Great efforts have been made in understanding *Cymbidium* biotechnology, particularly tissue culture and transgenics [1–4], and, together with *Phalaenopsis*, *Dendrobium* and *Cattleya*, *Cymbidium* is one of the most important orchids for the cut-flower and potted plant markets. Chinese *Cymbidium*, which holds a strong position in traditional flower markets both on mainland China and in Taiwan, is of great horticultural value as an ornamental plant because of its beautiful and fragrant flowers. Chinese *Cymbidium* includes *C. sinense*, *C. goeringii*, *C. forrestii*, *C. faberi*, *C. ensifolium* and *C. kanran. C. sinense* is a winter blooming epiphytic orchid usually regarded as a Spring Festival flower. *C. goeringii* is one of the most popular terrestrial species indigenous to temperate eastern Asia, cultivated as an ornamental, and whose flowers are used as ingredients of a soup, an alcoholic drink and tea [5]. *C. ensifolium* is a popular miniature terrestrial orchid which produces fragrant flowers and is often marketed as a potted specimen [6].

Chinese *Cymbidium* has a relatively long juvenile phase, taking more than 3 years to flower. Floral development in Chinese *Cymbidium* is regulated mainly by temperature and photoperiod, although it is still not clear how these pathways affect the genetics of flowering time. Cloning the genes involved in these pathways may elucidate their role during floral bud development. Very little research exists on the role of flowering genes in the regulation of the vegetative to flower transition and flower initiation in orchids, primarily in *Oncidium* [7,8]. No reports investigating the functions of flowering time genes of Chinese *Cymbidium* exist. *FLOWERING LOCUS T* (*FT*), a well-known floral integrator gene, plays an important role in controlling flowering time [9–13]. In the present study, we report on the isolation and functional analysis of *FT* gene orthologs from *C. sinense* “Qi Jian Bai Mo”, *C. goeringii* and *C. ensifolium* “Jin Si Ma Wei”. Ectopic expression of *CgFT*, which promoted flowering when transformed into *Arabidopsis*, has been demonstrated. Furthermore, we provide evidence that flowering time in transgenic *Arabidopsis* plants is altered due to the induction of the flower meristem identity gene *AP1*, as a result of the introduction and ectopic expression of the *CgFT* gene.

## 2. Results

### 2.1. Isolation of three FT cDNAs from *Cymbidium*

**Figure 1:**
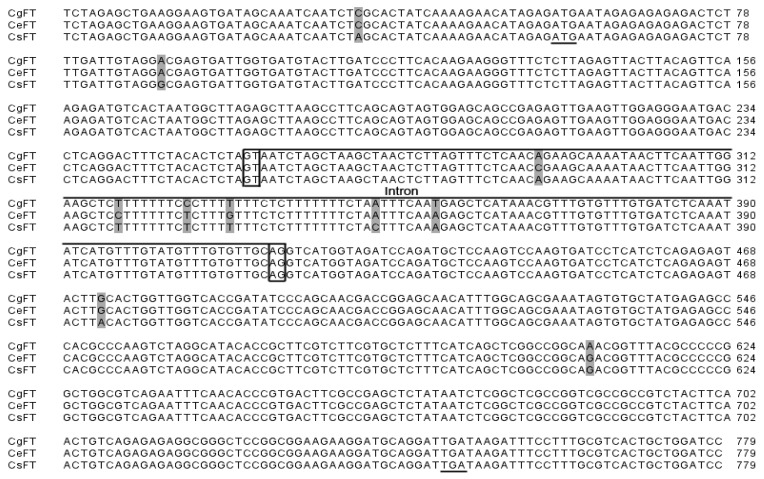
DNA sequence alignment of *CsFT*, *CgFT* and *CeFT.* Start and termination codons are underlined; The intron is marked by lines above the sequence; GT-AG are marked with open boxes; non-identical nucleotide acids are marked with shaded boxes.

**Figure 2:**
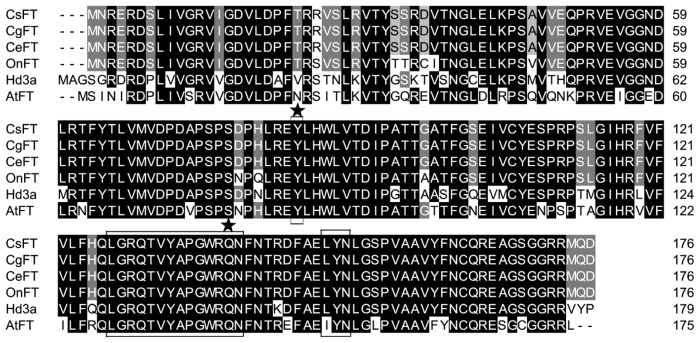
Multiple sequence alignment of amino acid sequence of *Cymbidium* FT with amino acid sequences of OnFT (*Oncidium* Gower Ramsey, Genebank accession number EU583502), Hd3a (*Oryza sativa* Japonica Group, Genebank accession number BAB61028.1), AtFT (*Arabidopsis thaliana*, Genebank accession number BAA77838.1). The black and shaded regions represent identical residues and conservative substitutions, respectively. The dots represent gaps inserted to optimize the alignment. Asterisks represent conserved domains.

**Figure 3:**
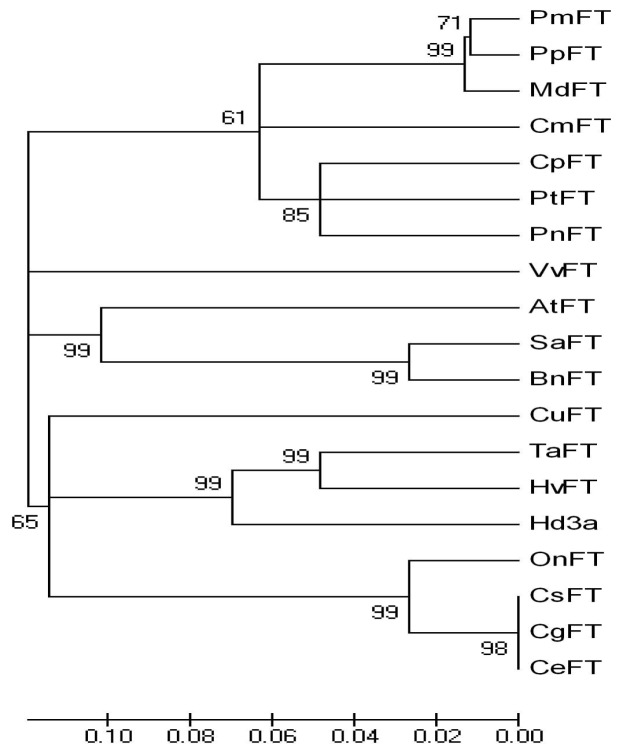
Phylogenetic analysis of the FT proteins from different plant species. OnFT (*Oncidium* Gower Ramsey, ACC59806.10); TaFT (*Triticum aestivum*, ACA25437.1); HvFT (*Hordeum vulgare*, ABJ97441.1); Hd3a (*Oryza sativa* Japonica Group, BAB61028.1); PnFT (*Populus nigra*, BAD02371.1); PtFT (*Populus trichocarpa*, XP_002334492.1); CpFT (*Carica papaya*, ACX85427.1); CuFT (*Citrus unshiu*, BAF96644.1); BnFT (*Brassica napus*, ACY03405.1); VvFT (*Vitis vinifera*, ABL98120.1); AtFT (*Arabidopsis thaliana*, BAA77838.1); PmFT (*Prunus mume*, BAH82787.1); PpFT (*Prunus persica*, ACH73165.1); SaFT (*Sinapis alba*, ACM69283.1); CmFT (*Cucurbita moschata*, ABR20499.1); MdFT (*Malus* × *domestica*, ACL98164.1). Bootstrap values were derived from 1000 replicate runs.

To investigate the role of the PEBP/RKIP gene family in regulating the transition from vegetative to reproductive growth in *Cymbidium*, PEBP orthologs were identified and characterized. A combined RT-PCR and RACE strategy was used to clone *FT* from *C. sinense* “Qi Jian Bai Mo”, *C. goeringii* and *C. ensifolium* “Jin Si Ma Wei”. *CsFT* (GenBank accession number HM120862), *CgFT* (GenBank accession number HM120863) and *CeFT* (GenBank accession number HM803115) contain 618-bp nucleotides with an open reading frame (ORF) of 531 bp encoding 176 amino acids (AAs), two exons and one intron (161 bp) (Figure 1). The analysis based on AA sequence alignment shows that the three FT AA sequences are identical; they also share a high identity with FT of other plants in GenBank, such as OnFT (94%) from *Oncidium* Gower Ramsey, Hd3a (79%) from *Oryza sativa*, and FT (74%) from *Arabidopsis thaliana* (Figure 2). AA sequence alignment also revealed that CsFT, CgFT and CeFT contain a conserved domain, which is characteristic of the PEBP-RKIP superfamily. The conserved key AA residues Tyr (Y, site 84) and Gln (Q, site 140) in FT homologs were identified in CsFT, CgFT and CeFT protein (Figure 2). The sequence similarity between CsFT, CgFT, CeFT and other FTs indicates that CsFT, CgFT, and CeFT are the putative *Cymbidium* FT orthologs. The conserved domains were analyzed in FT: LGRQTVYAPGWRQN (14 AAs) and LYN/IYN conserved domain was similar to other FT proteins [14]. The *Cymbidium* genes characterized here are closely related to the *FT* gene from monocotyledonous plants (*Oncidium* and *Oryza sativa*) [8,15] based on their protein sequences which have a conserved domain LYN (Figure 2). This suggests that CsFT, CgFT, and CeFT are potentially FT orthologs that regulate the transition from vegetative state to flowering and flower initiation in *Cymbidium*. The AA sequence alignment shown in Figure 2 and the sequences for several other FT orthologs were used to construct a phylogenetic tree for the *FT* group of genes (Figure 3). CsFT, CgFT and CeFT were grouped within the monocotyledonous FT subgroup and are closely related to *Oncidium*, followed by *Triticum aestivum*, *Hordeum vulgare* and *Oryza sativa*.

### 2.2. Different Expression Patterns of *Cymbidium FT* genes under LD/SD

**Figure 4:**
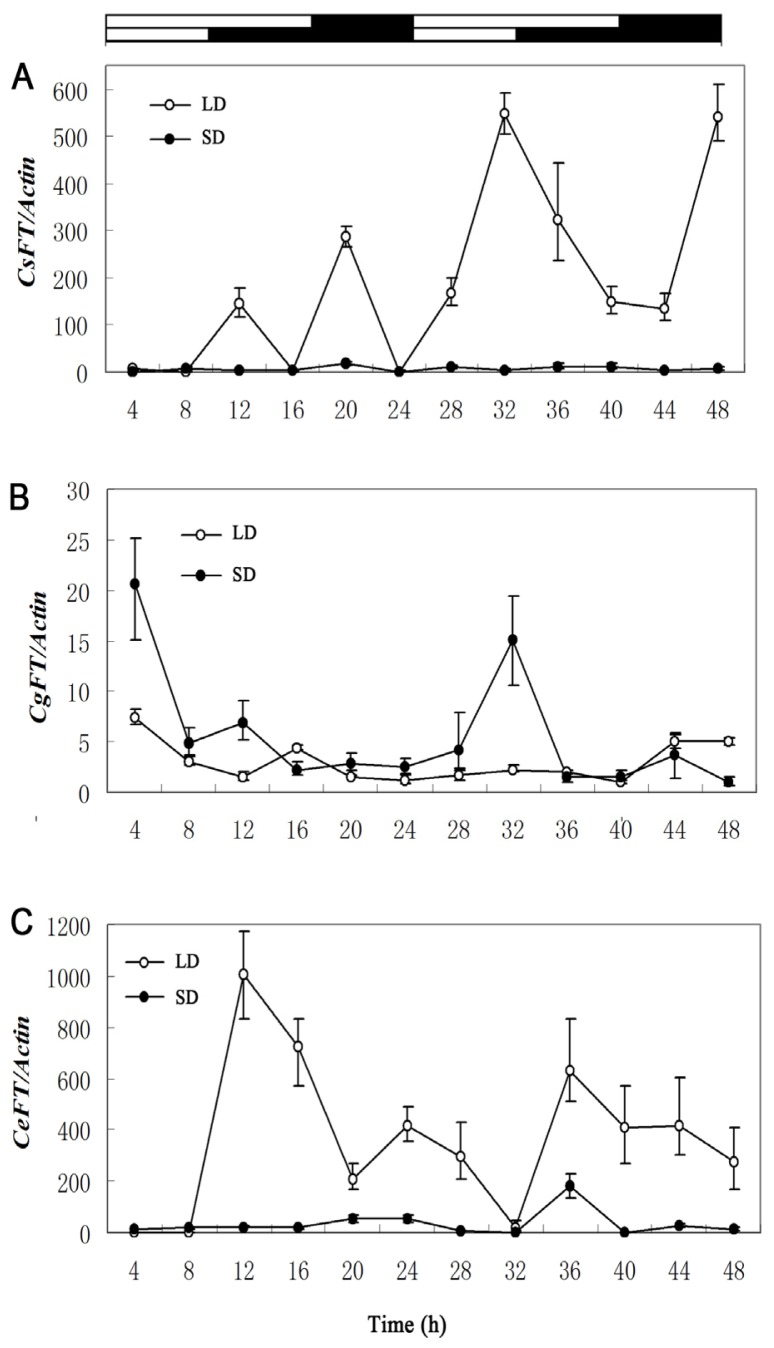
Diurnal expression pattern of *FT* in long days and short days. Expression pattern of (**A**) *CsFT* (*Cymbidium sinense*); (**B**) *CgFT* (*Cymbidium goeringii*); (**C**) *CeFT* (*Cymbidium ensifolium*). LD is marked by open circles. SD is marked by closed circles. Error bars depict SD (*n* = 4). The materials were measured using non-annual leaves of good condition.

To explore whether the expression of *CsFT*, *CgFT*, and *CeFT* was influenced by daily oscillations in photoperiod, the expression of *CsFT*, *CgFT*, and *CeFT* mRNA was analyzed every 4 h over a 24-h period in long day (LD) and short day (SD) conditions using quantitative real time PCR (Figure 4). *CsFT* expression (Figure 4A) was different under SD and LD: it was higher under LD than under SD. Expression of *CsFT* showed an increasing trend under LD, indicating that expression of *CsFT* was very sensitive to changes in light under LD. *CgFT* (Figure 4B) was also regulated by light but its expression was significantly higher under SD than under LD, but there was a similar change in pattern under both conditions, with the level highest at the 4th hour of the light period and lowest at the 16th hour. *C. ensifolium* “Jin Si Ma Wei” *CeFT* (Figure 4C) showed higher expression under LD than SD with a rhythmic cycle, but peaked at different times (12 h and 36 h of LD, 20 h and 36 h of SD). *CeFT* mRNA showed highest levels of expression 12 h into the light period and lowest levels at dawn. *CgFT* expression was thus different from *CsFT* and *CeFT* expression and all were regulated by light.

### 2.3. Ectopic Expression of *CgFT* Caused Early Flowering Phenotypes in *Arabidopsis Thaliana*

**Figure 5:**
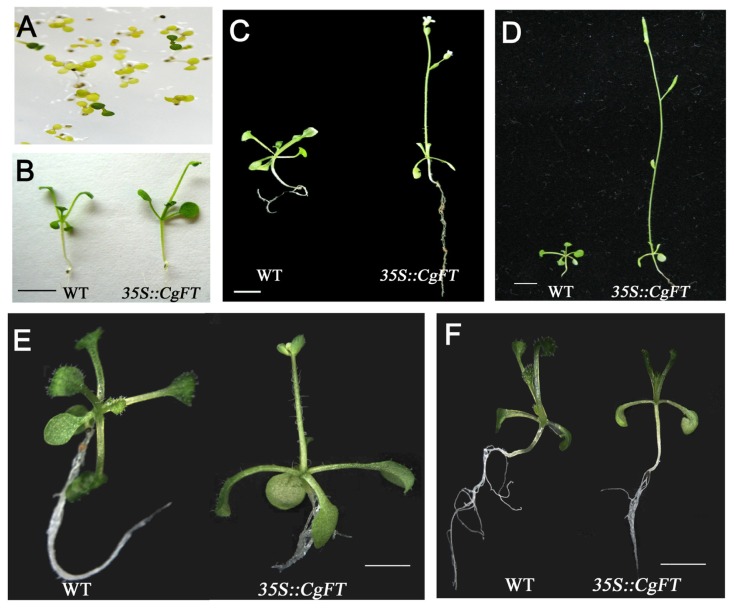
Phenotypic analysis of transgenic *Arabidopsis thaliana* plants (*35S::CgFT*) that ectopically expressed *CgFT*. (**A**) Screening transgenic *A. thaliana* T_0_ lines on 1/2 MS containing 50 μg/mL Kan. (**B**–**D**) Phenotype of WT and *35S::CgFT* plants (line 7-1, T_1_) at different stages of 12 h L/12 h D. The green lines of *A. thaliana* are putative transgenic plants that are (**B**) 15-days-old; (**C**) 20-days-old; (**D**) 25-days-old; (**E**) Phenotype of WT and *35S::CgFT* plants (line 7-3-5 T_2_) under 14 days long day (LD, 16 h L/8 h D) treatment; (**F**) Phenotype of WT and *35S::CgFT* plants (line 7-3-5 T_2_) under 14 days short day (SD, 8h L/16 h D) treatment. Bar = 5 mm for A, C, D, E, F.

Since the three *Cymbidium* FTs have the same AA sequences, we then randomly chose only one (*CgFT*) for functional verification. *CgFT* driven by the CaMV 35S promoter was transformed into wild-type (WT) *A. thaliana* plants for functional analysis in order to explore whether *Cymbidium FT* could regulate the transition to flowering in *A. thaliana. 35S::CgFT* transgenic *A. thaliana* T_0_ plants were screened on half-strength MS [16] containing 50 μg/mL Kan using a protocol from Cheng *et al*. [17] (Figure 5A). In total, 45 independent *35S::CgFT* transgenic *A. thaliana* T_1_ plants were obtained. All transgenic plants showed identical phenotypes by flowering earlier than WT plants (Figure 5B–D). These *35S::CgFT* transgenic plants (Figure 5) flowered at about 15 days after sowing by producing about four rosette leaves (Table 1; Figure 5B). The flowering time of WT *A. thaliana* plants was more than 30 days and produced nine or ten rosette leaves (Table 1). When transgenic plants were exposed to LD or SD, flowering was obviously promoted under LD (Figure 5E), but was slower under SD (Figure 5F) and ectopic expression of *cgFT* in *A. thaliana* was consistent with the *AtFT* gene expression pattern in *A. thaliana* [18,19].

**Figure 6:**
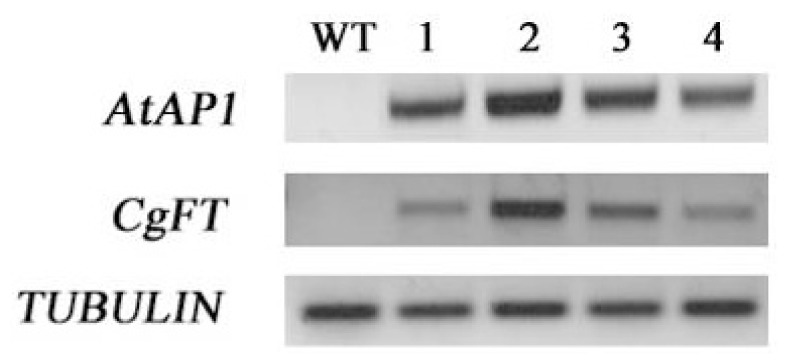
Expression analysis of related genes from *35S::CgFT* transgenic plants under 12 days LD (16 h L/8 h D) conditions. The amount of *TUBULIN* transcripts in *Arabidopsis thaliana* was used as an internal control. 1–4 represent four lines (3-4, 7-8, 30-10, 9-5) of *35S::CgFT* transgenic plants. The annealing temperature in PCR was 58 °C for *TUBULIN*, *CgFT* and *AtAP1.* 25 cycles for TUBULIN, 28 cycles for *CgFT* and *AtAP1*. The materials were measured using leaves of WT and transgenic *Arabidopsis thaliana* plants.

To explore whether the early flowering phenotype was correlated with *CgFT* expression in *35S::CgFT* transgenic plants, RT-PCR analysis was performed. As shown in Figure 6, higher *CgFT* expression was observed in the severe *35S::CgFT* transgenic plants more than in the transgenic plants with a less severe or WT phenotype. Further analysis indicated that the promotion of flowering time in severe early flowering *35S::CgFT* transgenic plants was also related to significant up-regulation of the flower meristem identity gene *AtAP1* in transgenic plants (Figure 6). This result indicates that the function of *Cymbidium FT* is similar to that of *A. thaliana FT* in regulating flowering time.

## 3. Discussion

### 3.1. Cloning and Characterization of three *FT* Homologous Genes from Chinese *Cymbidium*

Flowering time is regulated by a special set of flowering genes [20]. A number of studies indicated that in different plant species, *FLOWERING LOCUS T* (*FT*) homologous genes are involved in the earliest stages of flower development [21,22]. Our result shows that the structure of Chinese *Cymbidium FT* homologous genes contains two exons and one intron. This is not similar to other species, such as *A. thaliana* [10], *Oryza sativa* [23], *Hordeum vulgare* [24], *Vitis vinifera* [22], *Populus* sp. [25], and *Zea mays* [26], all of which have four exons and three introns. However, the wheat *TaFT* and barley *HvFT* genes have three exons encoding a 177-AA protein [27], in contrast with four exons and three introns in *HvFT2*, *HvFT3*, *HvFT4* and *HvFT5* of barley [24]. There is a loss of an intron in several barley cultivars, wild barley accessions, and wheat, suggesting that this may be a general feature of temperate grasses [24]. However, we deduced that the lack of introns cannot serve as a common feature of winter plants (*i.e.*, plants that require low temperature for vernalization) because *C. ensifolium* does not need low temperature for vernalization and has a long flowering period (April-October) in China. *C. goeringii*, however, needs low temperature for vernalization. For *C. sinense* “Qi Jian Bai Mo”, photoperiod has a major impact on flowering. The origins and importance of spliceosomal introns comprise one of the longest-abiding mysteries of molecular evolution [28]. The rates of loss of existent introns are higher than the rates of gain in eukaryotic evolution, and the rates of loss of introns represent the rate of evolution of a species [29]. It has been suggested that the process of intron birth in early eukaryotes could be fundamentally different from the process in more recent evolution [29]. Interestingly, the *Cymbidium FT* intron is also different from that of *Oncidium*, which has four exons and three introns [8]. The loss of an intron in the *Cymbidium FT* gene may be molecular evidence that *Cymbidium* is evolutionarily more advanced than *Oncidium*.

### 3.2. Expression Patterns and Regulation

*FT* gene expression increases dramatically when plants are induced by light [15,30]. Our results showed that expression of *CsFT* and *CeFT* were regulated by light and were significantly higher under LD than under SD, but that *CgFT* expression was significantly higher under SD. These results suggest that *C. goeringii* may be SD-sensitive plants regulated by light, similarly to *FT* in rice [15], while *C. sinense* “Qi Jian Bai Mo” and *C. ensifolium* “Jin Si Ma Wei” may be an LD-sensitive plant like *A. thaliana*. The difference in expression pattern of *Cymbidium FT* orthologs in response to light may be due to the fact that the *Cymbidium* genus contains species with different photoperiodic requirements, unlike *A. thaliana*, which is an LD plant, and rice, which is an SD plant. This pattern indicates that the expression of *CsFT* peaked at the 32nd hour of LD and *CeFT* expression peaked at the 12th hour of LD. However, this is unlike *At FT*, whose expression level peaks at the 16th hour of the light period and is lowest at dawn under LD conditions [18,19]. Interestingly, although the AA sequence of CsFT, CgFT and CeFT are identical, they have different expression patterns under LD and SD, indicating that expression of *CsFT*, *CgFT* and *CeFT* may also be affected by other factors, such as the upstream gene *CONSTANS* (*CO*)*-like* gene in the photoperiod pathway and other circadian clock genes [31], leading to diverse and complex *FT* expression patterns. The expression quantity of *CsFT* and *CeFT* are enormously higher than that of *CgFT* under LD, implying that *C. sinense* and *C. ensifolium* may primarily be regulated by photoperiod while *C. goeringii* may be mainly regulated by other factors, such as low temperature.

### 3.3. Constitutive Expression of CgFT Acts Similarly to *Arabidopsis Thaliana* FT in Regulating Flower Transition

FT plays a central role as a florigen in floral induction, and its function is conserved across different plant species. The transgenic plants carrying the *35S::CgFT* construct flowered earlier (about 23 days from sowing to flowering) and had fewer rosette leaves (average = 4.2) at flowering than the WT plants (average = 10.4). The early-flowering phenotype observed in *35S::CgFT* transgenic plants was similar to that observed in *A. thaliana* [9,10], *Solanum lycopersicum* [32], *Pharbitis nil* [33], *Vitis vinifera* [22], *Cucurbita moschata* [34], *Populus* sp. [25], *Oryza sativa* [35,36], *Oncidium* Gower Ramsey [7,8] and *Glycine max* [37] that ectopically express *FT* orthologs; in all cases, transgenic plants had a flowering time of less than 25 days and the number of rosette leaves at flowering was fewer than five. In contrast, flowering time in *A. thaliana* and rice plants could be delayed by using RNAi or miRNA of the *FT* gene [15,35]. Our results suggest that the function of the *FT* gene in flowering plants is very conservative and that *FT* and its homologs are required for flowering plants, regardless of photoperiod or species (dicotyledonous or monocotyledonous). Of importance, the level of the *AtAP1* transcripts was related to the expression of *CgFT* (Figure 6): the expression of *AtAP1* was higher when *FT* expression was higher. This indicates that constitutive expression of *CgFT* acts similarly to *AtFT* by regulating the transition from vegetative state to flowering by activating *AtAP1*.

## 4. Experimental Section

### 4.1. Plant Materials and Growth Conditions

*C. sinense* “Qi Jian Bai Mo”, *C. goeringii* and *C. ensifolium* “Jin Si Ma Wei” plants used in this study were grown and maintained in pots in a greenhouse of the South China Botanical Garden, Guangzhou, China. The pot substrate consisted of Zhijing stone for orchids (Northridge Enterprise Co., Ltd., Taiwan): sieved peat: shattered fir (1:1:1; *v*/*v*/*v*). *Arabidopsis thaliana* “Columbia” seeds obtained from our lab were surface sterilized in 70% ethanol for 10 s, then immersed in 0.1% (*w*/*v*) HgCl_2_ solution for 10 min followed by five rinses with sterile distilled water. *A. thaliana* seeds were sterilized and placed on agar plates containing half-strength Murashige and Skoog medium [16] at 4 °C for 2 days. The seedlings were then grown in a growth chamber under LD conditions (16-h light/8-h dark) or SD conditions (8-h light/16-h dark) at 22 °C for 10 days, then transplanted to soil under LD or SD conditions. The light intensity in the growth chambers was 150 μmol m^−2^ s^−1^.

### 4.2. Cloning *CsFT, CgFT* and *CeFT* cDNAs from *Cymbidium*

Total RNA was extracted from young leaves of all three *Cymbidium* spp. using the Trizol method. The first cDNAs strands were synthesized by reverse transcription PCR (RT-PCR) using M-MLV reverse transcriptase (TaKaRa Bio. Co., Ltd., Dalian, China). The primers (forward: 5′-TAGGACG AGTGATTGGTGA-3′; reverse: 5′-TCACTTGGACTTGGAGCAT-3′) designed for cDNA fragment amplification in PCR experiments were as described for the *OnFT* sequence from Genebank (ACC59806.1). The amplified fragments contained a partial *FT* sequence that showed high sequence identity to the *FT* homologous gene of other species from NCBI Blast. Gene-specific nested primers for 3′-RACE of *FT* were 5′-TAGGACGAGTGATTGGTGA-3′ (forward), 5′-TTCAGCAGTAGTGG AGCAG-3′ (reverse) designed using the cDNA fragment of *FT* from *Cymbidium*. Gene-specific primers (hiTAIL-PCR) [38] for 5′-RACE of *FT* were 5′-TGGAGCATCTGGATCTACCATGACCTG-3′, 5′-ACGATGGACTCCAGTCCGGCCGAAAGTCCTGAGGTCATTCCCTCCAC-3′, and 5′-CTCGGC TGCTCCACTACTGCTGAAGGC-3′, designed by using the cDNA fragment and the 3′-RACE of *FT* from *Cymbidium*. The cDNAs and genomic DNA sequences for the three *FT* genes were obtained by PCR amplification using forward (5′-CTGAAGGAAGTGATAGCAA-3′) and reverse (5′-AGCAGT GACGCAAAGGAAA-3′) primers designed by using the full-length sequence of *FT* from *Cymbidium* after 5′-RACE. The forward primer for *FT* contained an *Xba*I recognition site (5′-TCTAGA-3′) and the reverse primer contained a *BamH*I recognition site (5′-GGATCC-3′) to facilitate the cloning of the cDNAs.

### 4.3. Real-Time PCR Analysis

Quantitative real-time PCR was carried out using SYBR^®^ Premix Ex Taq™ II (TaKaRa) and ABI 7500 real-time PCR for transcript measurements. Amplification conditions were: 95 °C for 30 s, followed by 40 cycles of amplification (95 °C for 5 s, 60 °C for 34 s, 72 °C for 30 s) and plate reading after each cycle. The primers designed from the full-length sequence of *FT* from *Cymbidium* used for quantitative real-time PCR for *CsFT*, *CgFT* and *CeFT* were: forward (5′-AGAGTTGAAG TTGGAGGGAATG-3′) and reverse (5′-GGTCGTTGCTGGGATATCG-3′). The primers for *Cymbidium ACTIN* (designed using the *CsActin* sequence, Genebank accession number GU181353) were: forward (5′-AATCCCAAGGCAAACAGA-3′) and reverse (5′-CCATyACCAGAATCCAG-3′). Data were analyzed using ABI 7500 Real-time PCR system Gene Expression software.

### 4.4. Phylogenetic Analysis

A phylogenetic comparison of aAA sequences of different FT-like homologs was performed using GenBank [39] and aligned with Clustal W2 [40]. Phylogenetic trees based on the complete sequences were generated using MEGA4 [41] and constructed by the neighbor joining (NJ) method. Bootstrap values were derived from 1000 replicate runs.

### 4.5. Semi-Quantitative RT-PCR

Total RNA was extracted from young leaves using TRIzol Reagent (Invitrogen, Carlsbad, CA, USA) following the manufacturer’s protocol. Each RNA sample was treated with RNase-free DNase (Promega, Madison, WI, USA) following the manufacturer’s protocol in an effort to remove any residual genomic DNA (gDNA). DNase-treated RNA was subjected to reverse transcriptase reactions using oligo-dT primer and PrimeScript™ Reverse Transcriptase (TaKaRa) according to the manufacturer’s protocol. The gene-specific primers (designed by using the full-length sequence of *FT* from *Cymbidium*) for *FT* used in all RT-PCR were 5′-TAGGACGAGTGATTGGTGA-3′ (forward) and 5′-TCACTTGGACTTGGAGCAT-3′ (reverse). The cDNA sequence of the *TUBULIN* gene in *A. thaliana* was used as a control and amplified using two primers: 5′-GAGCCTTACAACGCTA CTCTGTCTGTC-3′ (forward) and 5′-ACACCAGACATAGTAGCAGAAATCAA-3′ (reverse) [42]. The primers for *A. thaliana AtAP1* used in RT-PCR were 5′-GCACCTGAGTCCGACGTC-3′ (forward) and 5′-GCGGCGAAGCAGCCAAGG-3′ (reverse). PCR was performed with a PCR System LABCYCLER Standard Plus (SENSOQUEST, Hannah, Germany). The following thermocycling conditions were applied: initial denaturation at 94 °C for 1 min; 25 cycles of 94 °C for 30 s, 58 °C for 45 s, and 72 °C for 1 min; final extension at 72 °C for 10 min. The amplified products were separated on a 1.5% agarose gel using 0.1% ethidium bromide (EtBr) and photographed in a Bio Sens SC 710 system.

### 4.6. Plant Transformation and Transgenic Plant Analysis

The full-length cDNA for *CgFT* was cloned into the binary vector pBI121 (BD Biosciences, Clontech, San Jose, CA, USA) under the control of the CaMV 35S promoter. The orientation of the construct was determined by PCR and used for further plant transformation. *Arabidopsis* plants were transformed using a floral dip method [43]. Transformants that survived in medium containing kanamycin (50 μg mL^−1^) were further verified by RT-PCR analyses.

## 5. Conclusions

The three Chinese *Cymbidium* species in this study blossom in different seasons when they are planted in South China. *C. sinense* blossoms from November to February, *C. goeringii* from January to March and *C. ensifolium* from June to October. In this study, we present for the first time, the molecular characterization of *FT* homologs in their three Chinese *Cymbidium* species. Amino acid sequence analysis indicated high sequence identity with other FT sequences in other plants. Light period had a profound effect on transcript levels. Ectopic expression of *CgFT* (the *FT* ortholog of *Cymbidium goeringii*) in transgenic *Arabidopsis* plants resulted in early flowering compared to wild-type plants and significant up-regulation of *APETALA1* (*AP1*) expression. CgFT is a putative phosphatidylethanolamine-binding protein gene in *Cymbidium* that regulates the vegetative to reproductive transition in flowers. This is important data that sheds light on the molecular mechanisms of flowering in *Cymbidium*, which is an undocumented phenomenon to date.

## Figures and Tables

**Table 1:** Over-expression of *FT* in *Arabidopsis thaliana*. Bolting time, anthesis time and leaf number of wild-type, *35S::CgFT* transgenic T_1_ plants under LD (16 h L/8 h D) conditions.

Genotype	No. of plants	Days to bolting	Rosette leaf number at bolting	Days to anthesis	Rosette leaf number at anthesis
WT	20	22.7 ± 3.2	8.1 ± 1.9	30.4 ± 5.0	10.4 ± 2.1
*35S::CgFT* (line 7)	22	13.0 ± 1.5 **	4.0 ± 0.0 **	22.7 ± 3.3 **	4.2 ± 0.6 **
*35S::CgFT* (line 30)	13	14.1 ± 2.0 **	4.0 ± 0.0 **	23.8 ± 3.9 **	4.2 ± 0.6 **
*35S::CgFT* (line 9)	12	16.5 ± 3.7 **	4.3 ± 0.8 **	26.6 ± 3.1 *	4.8 ± 1.0 **

Values are the means ± SD from individual plants.

** and * indicate significant differences at *p* < 0.01 and *p* < 0.05 according to the *t*-test compared to wild type.
